# Emerging Roles of the Mitogen and Stress Activated Kinases MSK1 and MSK2

**DOI:** 10.3389/fcell.2016.00056

**Published:** 2016-06-10

**Authors:** Kathleen M. S. E. Reyskens, J. Simon C. Arthur

**Affiliations:** Division of Cell Signalling and Immunology, School of Life Sciences, University of DundeeDundee, UK

**Keywords:** MSK1, MSK2, CREB, MAPK, p38, IL-10, innate immunity, synaptic plasticity

## Abstract

Mitogen- and stress-activated kinases (MSK) 1 and 2 are nuclear proteins activated downstream of the ERK1/2 or p38 MAPK pathways. MSKs phosphorylate multiple substrates, including CREB and Histone H3, and their major role is the regulation of specific subsets of Immediate Early genes (IEG). While MSKs are expressed in multiple tissues, their levels are high in immune and neuronal cells and it is in these systems most is known about their function. In immunity, MSKs have predominantly anti-inflammatory roles and help regulate production of the anti-inflammatory cytokine IL-10. In the CNS they are implicated in neuronal proliferation and synaptic plasticity. In this review we will focus on recent advances in understanding the roles of MSKs in the innate immune system and neuronal function.

## Introduction

MSK1 and 2 were first identified in 1998 as proteins that shared homology to the RSK kinase family (Deak et al., 1998; New et al., 1998; Pierrat et al., 1998). MSKs contain 2 kinase domains—an N-terminal kinase domain (NTKD) in the AGC kinase family and a C-terminal kinase domain (CTKD) from the calmodulin kinase family (Caenepeel et al., 2004). MSKs are activated by the ERK1/2 or p38 MAPK pathways. ERK1/2 and/or p38 phosphorylates 3 sites on MSKs, which activates the CTKD. This causes autophosphorylation and activation of the NTKD, which in turn phosphorylates MSK substrates (Deak et al., 1998; McCoy et al., 2005, 2007). MSKs exist in all vertebrate species examined, with the exception of the lamprey. The chordates *Ciona intestinalis, Ciona savignyi*, and *Branchiostoma floridae* possess a single MSK homolog indicating that MSKs diverged from RSK before the onset of vertebrate evolution. MSK1 and 2 probably derive from a duplication event early in vertebrate evolution; orthologs of both MSK1 and 2 exist in the elephant shark, *Callorhinchus milii* (Venkatesh et al., 2014) and in bony fish. In cells, MSK1 and MSK2 are functionally redundant (Wiggin et al., 2002). Perhaps because of this, not all vertebrates have retained both genes. In the Ensembl database, MSK2 is absent in 5 bird genomes while 11 out of the 38 mammalian genomes appear to have lost MSK isoforms. Orthologs of MSKs have been identified in nematodes and insects, although of these only the *Drosophila* protein Jil-1 has been studied in detail (Jin et al., 1999; Wang et al., 2001). Interestingly the main area of homology for Jil-1 with MSKs is in the NTKD. Unlike the chordate and nematode MSKs, the CTKD of Jil-1 lacks the classical MAPK phosphorylation sites.

MSKs are predominantly localized to the nucleus and this is reflected in their known substrates. The best characterized MSK substrates are Histone H3 and the related transcription factors CREB and ATF1. These have been validated via both pharmacological inhibition and mouse genetics (reviewed in Arthur, 2008). The majority of the genes regulated by MSKs are also targets of CREB. The detailed molecular mechanism by which MSKs regulate CREB is however unclear. MSKs phosphorylate CREB on Ser133, a site that is also targeted by other kinases including protein kinase A (PKA). Phosphorylation of CREB by PKA creates a binding site for the co-activator proteins CBP and p300 (Gonzalez and Montminy, 1989; Chrivia et al., 1993; Cardinaux et al., 2000; Mayr and Montminy, 2001), whose recruitment promotes the efficient transcription of CREB target genes (Yamamoto et al., 1988; Gonzalez and Montminy, 1989). Several studies have demonstrated that CREB phosphorylation downstream of MAPK signaling does not lead to CBP or p300 recruitment (Brindle et al., 1995; Mayr and Montminy, 2001; Mayr et al., 2001; Kasper et al., 2011), leading to the suggestion that MAPKs, and by inference MSKs, do not activate CREB-dependent transcription. Furthermore, PKA- but not MSK-mediated CREB phosphorylation leads to efficient CBP or p300 recruitment to endogenous CREB-dependent promoters as judged by chromatin immunoprecipitation (ChIP) (Naqvi et al., 2014). Despite this, mutation of the Ser133 site to alanine in the endogenous CREB gene actually had a bigger impact on CREB target genes in response to MSK activating stimuli compared to PKA activating stimuli. How MSK-mediated CREB phosphorylation activates CREB is however unresolved (Naqvi et al., 2014). A number of other substrates for MSKs, including NFκB, HMG-14, RAR-related orphan receptor alpha (RORα), KDM3A, Trim7, and Trim28 have been proposed, although their overall importance to MSK function is currently less clear (Soloaga et al., 2003; Vermeulen et al., 2003; Bruck et al., 2009; Cheng et al., 2014; Chakraborty et al., 2015; Singh et al., 2015). While MSKs are expressed in many tissues, their function, as discussed below, has been best studied in the innate immune system and brain.

### MSKs in innate immunity

p38α MAPK has been extensively studied in innate immunity and regulates the production of pro-inflammatory cytokines in innate immune cells. As a result p38 inhibitors have been developed as potential anti-inflammatory drugs, although none have progressed in the clinic. More recently, anti-inflammatory roles for p38 have emerged which may contribute to the lack of efficacy of p38 inhibitors in the clinic (reviewed in Arthur and Ley, 2013; Salgado et al., 2014). A key question therefore was whether MSKs regulated pro- or anti-inflammatory functions downstream of p38α. MSK1/2 knockout does not result in an overt phenotype in unchallenged mice (Wiggin et al., 2002), however these mice are sensitized to lipopolysaccharide (LPS)-induced endotoxic shock, indicating that MSK activation has an anti-inflammatory effect (Ananieva et al., 2008). MSK1/2 knockout also resulted in elevated levels of TNF, IL-6, and IL-12 production downstream of LPS stimulation, but decreased production of the anti-inflammatory cytokine IL-10 (Ananieva et al., 2008; Kim et al., 2008). IL-10 is known to repress pro-inflammatory cytokine production, and neutralization of IL-10 in wildtype isolated macrophages increases the production of IL-6 and IL-12, similar to that found in MSK1/2 knockouts with decreased endogenous IL-10. However, additional neutralization of IL-10 in MSK1/2 knockouts did not further affect pro-inflammatory cytokine production in isolated macrophages (Ananieva et al., 2008). Interestingly the effect on TNF was partial, suggesting additional IL-10 independent mechanisms of TNF regulation by MSKs.

**Figure 1:**
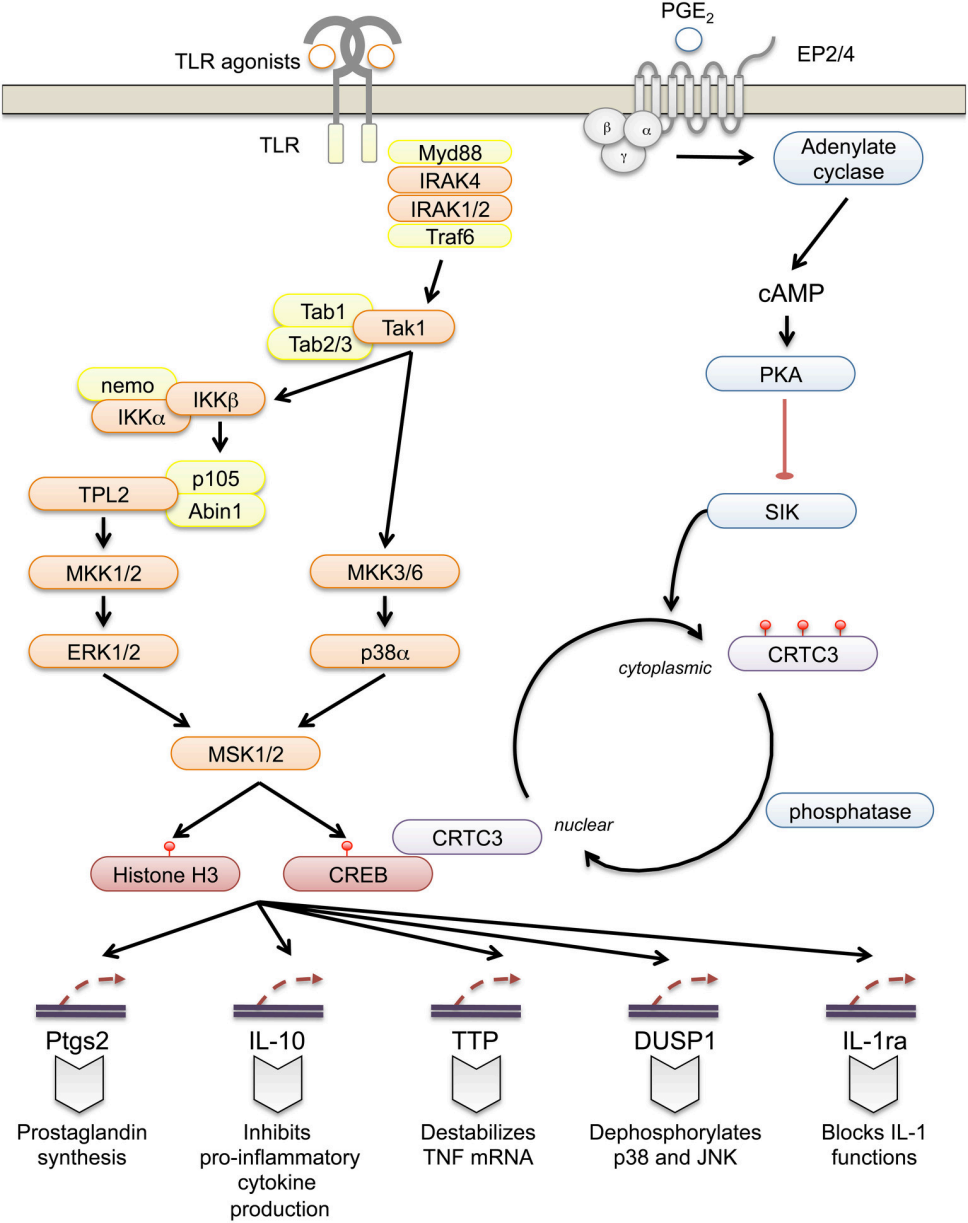
**Regulation of innate immune function by MSK1 and 2**. In innate immune cells such as macrophages and dendritic cells TLRs, with the exception of TLR3, can activate downstream signaling via Myd88. Upon ligand binding, the TLR recruits Myd88 resulting in the formation of a Myd88osome that also contains IRAK4 and IRAK1 and/or 2. This leads to the recruitment of Traf6 and the formation of K63 and M1 ubiquitin chains that help mediate the activation of Tak1. Tak1 then activates the p38 MAPK cascade and indirectly activates ERK1/2 via signaling to the IKK-mediated activation of Tpl2 through p105. ERK1/2 and p38 are then both able to activate MSK1 and 2, which in turn phosphorylate CREB and Histone H3. This leads to the induction of a number of genes with potential anti-inflammatory roles including *IL-10, IL-1ra, TTP, DUSP1*, and *Ptgs2*. Ptgs2 is the rate-limiting enzyme in the production of prostaglandins, small lipids that can have both pro- and anti-inflammatory roles. In the context of macrophages, prostaglandin E can boost IL-10 production and suppress pro-inflammatory cytokines following TLR stimulation. It acts via the G-protein coupled receptors EP2 and EP3 to elevate cAMP levels and activate PKA. PKA phosphorylates SIK2 and inhibits its ability to phosphorylate CRTC3. This allows CRTC3 to be dephosphorylated and translocated to the nucleus where it will act as a co-activator for CREB.

MSKs regulate IL-10 by controlling its transcription in macrophages and dendritic cells (Ananieva et al., 2008; Elcombe et al., 2013). In addition to IL-10, several other anti-inflammatory genes are regulated by MSKs in macrophages, including dual specificity protein phosphatase 1 (DUSP1), tristetraprolin (TTP) and IL-1 receptor agonist (IL-1ra) (Brook et al., 2006; Ananieva et al., 2008; Darragh et al., 2010) (Figure 1). DUSP1 is a phosphatase that inactivates the p38 and JNK MAPKs, and like MSK1/2 knockout, DUSP1 knockout sensitizes mice to endotoxic shock (reviewed in Lang et al., 2006; Wang and Liu, 2007). TTP is able to bind to AU rich elements in the 3′ UTR of multiple cytokines including TNF, inhibiting translation and promoting mRNA degradation (Brooks and Blackshear, 2013). IL-1ra is a member of the IL-1 family that binds to the IL-1 receptor but cannot activate signaling, thus acting as an inhibitor of IL-1 *in vivo* (Garlanda et al., 2013).

TTP knockout gives rise to multiple inflammatory phenotypes leading to early mortality (Taylor et al., 1996). Loss of IL-10 in mice or humans results in the development of colitis (Kühn et al., 1993; Shah et al., 2012) while mutation of IL-1ra in humans gives rise to an early onset auto-inflammatory condition, DIRA (Aksentijevich et al., 2009). Given the phenotypes associated with its target genes, it is surprising that MSK knockout does not give a more overt phenotype. This may be because other pathways compensate for MSK *in vivo*. Related to this, CREB is phosphorylated by other kinases in addition to MSKs including PKA (Johannessen et al., 2004). Prostaglandin E2 (PGE_2_) activates PKA in macrophages and acts synergistically with TLR agonists to induce IL-10. This effect is dependent on CREB, but does not require PKA-mediated CREB phosphorylation (MacKenzie et al., 2013b). Instead PKA phosphorylates salt-inducible kinase 2 (SIK2) thus inhibiting the ability of SIK2 to phosphorylate CRTC3. As a result CRTC3 becomes dephosphorylated and translocates to the nucleus where it acts as a co-activator for CREB on the IL-10 promoter (Clark et al., 2012; MacKenzie et al., 2013b).

MSKs also have complex roles in regulating prostaglandin production. *Ptgs2*, the rate-limiting enzyme in prostaglandin production, is a CREB regulated gene. Early *Ptgs2* transcription downstream of TLR agonists is positively regulated by MSKs, suggesting that MSK inhibition might reduce prostaglandin production. IL-10 can however suppress *Ptgs2* mRNA induction. As MSK knockout reduces IL-10 production, at later time points after LPS stimulation MSK1/2 knockout macrophages actually show increased *Ptgs2* induction and elevated prostaglandin production (MacKenzie et al., 2013a).

In addition to their anti-inflammatory roles, various pro-inflammatory roles have also been suggested for MSKs. In human neutrophils LPS can induce MSK-dependent CREB phosphorylation. Both CREB phosphorylation and induction of TNF, IL-8 (CXCL8), CCL3 and CCL4 were reduced by Ro318220, an inhibitor that targets MSKs amongst other AGC kinases. In support of a role for CREB, transfection of PLB-985 cells, a neutrophil-like cell line, with a dominant negative CREB reduced IL-8 and TNF induction (Mayer et al., 2013). MSK1 has also been linked to IL-8 production in human keratinocytes and airway smooth muscle cells, where siRNA-mediated knockdown of MSK1 reduced IL-8 production (Funding et al., 2006; Rahman et al., 2014). Interestingly both MSK1 and 2 activity are increased in human lesional psoriatic skin (Funding et al., 2006, 2007), suggesting a potential involvement of MSKs in the pathology of this disease. Related to this, DMF is used as a treatment for psoriasis and it has been proposed that its efficacy may be in part due to MSK inhibition (Gesser et al., 2007; Peng et al., 2012). In mice however MSK1/2 knockout increases skin inflammation following PMA treatment or in oxazolone-induced allergic contact dermatitis (Ananieva et al., 2008; Bertelsen et al., 2011). This may reflect a difference between human and mouse skin; if in human keratinocytes MSK acts predominantly to regulate inflammation via IL-8, these differences may relate to the lack of a direct IL-8 homolog in mice.

*Herpesviridae* are a family of double stranded DNA viruses some of which, including Kaposi's sarcoma-associated herpes virus (KSHV) and human cytomegalovirus (HCMV), cause human disease. Upon infection these viruses can either enter a lytic replication cycle to produce further virions or enter a latent phase (Roizman and Baines, 1991). Following infection of Human Umbilical Vein Endothelial Cells (HUVEC) with KSHV, MSK is activated and phosphorylates CREB. siRNA-mediated knockdown of either CREB or MSK1 and 2 did not prevent infection, but did reduce the production of infectious virions. This correlated with a drop in the levels of the viral genes involved in lytic replication (Cheng et al., 2015). HCMV can be reactivated in cells with a latent infection via a process stimulated by IL-6 (Hargett and Shenk, 2010; Reeves and Compton, 2011). This process correlates with CREB phosphorylation and furthermore CREB binding can be demonstrated on the viral MIEP during reactivation (Kew et al., 2014). This process was blocked by inhibition of the ERK1/2 pathway, suggesting a role for either MSK or RSK. The RSK inhibitor DI-D1870 did not affect this process while H89, a compound that targets several kinases including MSKs, reduced transcription from the MIEP promoter (Kew et al., 2014).

### MSKs and neuronal function

Both MSK1 and MSK2 are expressed in the brain; however MSK1 is the major isoform in most brain structures (Arthur et al., 2004). While MSK1/2 knockout mice do not exhibit gross defects in CNS development, under some circumstances MSKs play roles in neuronal proliferation or survival. Following pilocarpine-induced seizure, proliferation of neuronal progenitors in the subgranular zone (SGZ) of the dentate gyrus of mice was reduced by knockout of MSK1 and 2. In addition reduced neurite arborization was also observed in immature neurons in this region, suggesting that MSKs were helping drive proliferation and the maturation of new neurons (Choi et al., 2012). Similar results were also reported in the SGZ in a model of cerebral ischemia (Karelina et al., 2015).

Several reports have also suggested roles for MSKs in neurodegenerative diseases. Spinocerebellar ataxia type 1 (SCA1), a condition resulting from the expansion of a polyglutamine tract in ataxin-1, results in neurodegeneration in the cerebellum and brain stem (Manto, 2005). Ataxin-1 can be phosphorylated by MSKs on Ser766 and lead to stabilization of mutant forms of the protein. Loss of one or more MSK alleles was protective in a mouse model of SCA1 driven by an Atxn^154*Q*^ mutation, raising the possibility that MSK inhibitors may be useful for treating this disease (Park et al., 2013). Huntington's disease results from the expansion of a CAG motif in the Htt gene giving rise to a polyglutamine repeat (Walker, 2007). While it affects many areas of the brain, the striatum is particularly sensitive to damage. Decreased MSK1 expression was observed in the caudate nucleus from the striatum of Huntington's patients (Roze et al., 2008). In the R6/2 transgenic mouse model of Huntington's, both MSK1 and Histone H3 Ser10 phosphorylation were decreased (Roze et al., 2008). Furthermore, MSK1 overexpression can promote expression of PGC-1α, a gene that is neuro-protective in Huntington's and MSK1 knockout mice showed evidence of striatal degeneration upon aging (Martin et al., 2011). Parkinson's disease is associated with reduced dopamine levels. Dopamine therapy, while beneficial for Parkinson's can result in Levodopa-induced dyskinesia (LID). In animal models this correlates with increased δFosB expression in the striatum. Two studies showed that MSK knockout decreased δFosB expression in LID, however while in one study LID intensity was attenuated in the other it was not (Brami-Cherrier et al., 2005; Alcacer et al., 2014; Feyder et al., 2016).

The striatum is also involved in addiction. MSK1 regulates glutamate-stimulated Histone H3 phosphorylation in cultured striatal neurons and cocaine administration activated MSK1 in the striatum *in vivo* (Brami-Cherrier et al., 2007). MSK1 knockout mice showed increased response to low doses of cocaine in placed preference tests, while following repeated injections of cocaine locomotor sensitization was decreased (Brami-Cherrier et al., 2005).

The hippocampus plays important roles in encoding memory and is involved in rodent models of spatial memory and contextual fear conditioning. CREB is implicated in synaptic plasticity and memory (Shaywitz and Greenberg, 1999; Lonze and Ginty, 2002; Carlezon et al., 2005; Benito and Barco, 2010; Sakamoto et al., 2011) while the importance of histone modifications, including phosphorylation, have also been recognized (Day and Sweatt, 2011; Mifsud et al., 2011; Kandel et al., 2014; Alberini and Kandel, 2015). The MAPK pathway has been also found to be a critical component of consolidation of memory in the hippocampus (Besnard et al., 2014) and ERK1/2-stimulated histone phosphorylation and acetylation in hippocampal CA1 neurons is associated with memory consolidation (Levenson et al., 2004; Chwang et al., 2006). The involvement of both the upstream activators of MSK and its substrates in these processes suggest the involvement of MSKs in memory.

In primary cortical neuronal cultures stimulated with the neurotrophin BDNF, MSK1 was the critical isoform for both CREB phosphorylation and the induction of CREB-dependent IEGs (Arthur et al., 2004). The effect of MSK1 knockout has now been reported in several hippocampal-dependent learning models. In the forced swim test, mice repeatedly placed in a pool with no ability to escape will display a learned helplessness (De Pablo et al., 1989; West, 1990; Korte, 2001). These behavioral changes are accompanied by an increase in phosphorylation and acetylation of Histone H3 in dentate gyrus granule neurons (Bilang-Bleuel et al., 2005), and knockout of MSK1/2 restricted this behavioral response and correlated with decreased Histone H3 phosphorylation (Chandramohan et al., 2008).

In contextual fear conditioning mice learn to associate a specific context with an aversive stimulus. MSK1 activation has been shown to occur during this process (Sindreu et al., 2007), which is also associated with CREB and Histone H3 phosphorylation (Impey et al., 1998; Taubenfeld et al., 1999). MSK1 knockout resulted in a mild deficit in contextual fear conditioning and decreased CREB and Histone H3 phosphorylation (Chwang et al., 2007). MSK1 knockout also resulted in a mild impairment in the Morris water maze (Chwang et al., 2007), a widely used model for spatial memory (D'Hooge and De Deyn, 2001). Long term potentiation (LTP), which allows the strengthening of specific synapses in response to high frequency stimulation, is a form of synaptic plasticity considered to provide a molecular model for encoding memory in the hippocampus (Martin et al., 2000). The role of MSKs have not been directly examined in LTP and it would be of interest to address this in the future. Related to this, in response to neurotrophins, MSKs control the production of two closely related miRNAs, miR-132, and miR-212 that have been implicated in synaptic function (Wayman et al., 2008; Remenyi et al., 2010). mir-132/miR-212 knockouts showed normal LTP in the hippocampus but impaired neocortical theta burst-induced LTP (Remenyi et al., 2013).

Although the role of MSKs has not been reported in LTP, their role has been investigated in homeostatic scaling. This is a form of non-Hebbian plasticity that allows a neuron to co-ordinately regulate the strengths of all its synaptic inputs in order to maintain its firing rate within physiological boundaries (Davis, 2006), thus allowing protection of neural networks from excessive or inhibitive stimuli. This scaling process has been linked to levels of BDNF (Rutherford et al., 1998; Turrigiano et al., 1998). Homeostatic scaling can be modeled in culture by globally blocking axon potentials using TTX, which blocks voltag- gated sodium channels. Cultured hippocampal neurons from MSK1 kinase-dead mice failed to show homeostatic scaling of synaptic transmission in response to TTX (Corrêa et al., 2012). BDNF is also involved in the synaptic scaling and remodeling that occurs in response to environmental enrichment *in vivo* (Baroncelli et al., 2010; Cowansage et al., 2010). A failure to upregulate synaptic strength during environmental enrichment has been reported in MSK1 kinase dead knockin (Corrêa et al., 2012) and MSK1 knockout mice (Sakamoto et al., 2011). Given the role of MSKs in response to environmental enrichment it will be especially interesting to look at the effect of MSK knockout on LTP and memory in animals raised in enriched conditions.

## Conclusion

This review highlights the critical importance of MSKs in limiting inflammation in innate immunity and their role in inflammatory disease, however their role in the adaptive immune responses remain uncharacterized. MSKs are also implicated in neurodegeneration and synaptic plasticity. Currently, much more work remains to uncover their precise roles in these processes of inflammation and neurodegenerative disease. Outside these systems, work has recently suggested further roles for MSKs. For example MSKs may be involved in skin tumor formation (Chang et al., 2011; Dong et al., 2014; Liu et al., 2014) and have been linked to cellular transformation (Raab-Traub, 2002; Pérez-Cadahía et al., 2011; Reyes et al., 2014). Future research will help unravel the exact mechanisms underlying these enzymes *in vivo* in health and disease.

## Author contributions

All authors listed, have made substantial, direct and intellectual contribution to the work, and approved it for publication.

## Funding

SA is funded by the Medical Research Council and Arthritis Research UK.

### Conflict of interest statement

The authors declare that the research was conducted in the absence of any commercial or financial relationships that could be construed as a potential conflict of interest.
